# Is Universal Screening Necessary? Incidence of Tuberculosis among Tibetan Refugees Arriving in Calgary, Alberta

**DOI:** 10.1155/2016/8249843

**Published:** 2016-12-29

**Authors:** Rachel Lim, Julie Jarand, Stephen K. Field, Dina Fisher

**Affiliations:** ^1^Cumming School of Medicine, University of Calgary, Calgary, AB, Canada; ^2^Division of Respirology, Department of Medicine, University of Calgary, Calgary, AB, Canada

## Abstract

*Background*. Canadian policy requires refugees with a history of tuberculosis (TB) or abnormal chest radiograph to be screened after arrival for TB. However, Tibetan refugees are indiscriminately screened, regardless of preimmigration assessment. We sought to determine the incidence of latent (LTBI) and active TB, as well as treatment-related outcomes and associations between preimmigration factors and TB infection among Tibetan refugees arriving in Calgary, Alberta.* Design*. Retrospective cohort study including Tibetan refugees arriving between 2014 and 2016. Associations between preimmigration factors and incidence of latent and active TB were determined using Chi-square tests.* Results*. Out of 180 subjects, 49 percent had LTBI. LTBI was more common in migrants 30 years of age or older (*P* = 0.009). Treatment initiation and completion rates were high at 90 percent and 76 percent, respectively. No associations between preimmigration factors and treatment completion were found. A case of active TB was detected and treated.* Conclusion*. Within this cohort, the case of active TB would have been detected through the usual postsurveillance process due to a history of TB and abnormal chest radiograph. Forty-nine percent had LTBI, compared to previously quoted rates of 97 percent. Tibetan refugees should be screened for TB in a similar manner to other refugees resettling in Canada.

## 1. Introduction

Increasing globalization and population mobility call for joint efforts among developed and developing countries aimed at the elimination of tuberculosis (TB). More than half of active TB cases in Canada occur in foreign-born individuals, particularly those originating from TB endemic countries [1]. In 2012, immigrants and refugees accounted for 64 percent of reported cases of active TB in Canada [1]. Most cases of active TB are due to reactivation of latent tuberculosis infection (LTBI), rather than primary infection [2]. Refugees are at higher risk than immigrants due to both a higher prevalence of LTBI and crowded living conditions such as refugee camps [1]. Additional risk factors include country of origin, comorbidities such as chronic kidney disease or malignancy, and time since arrival, likely due to an acquisition of infection prior to departure [3].

Citizenship and Immigration Canada (CIC) regulations currently state that those with a previous history of TB or an abnormal prearrival chest radiograph must be assessed within 30 days of arrival in Canada for postlanding surveillance. The CIC implements this mandatory referral process for the sole purpose of identifying those with active TB at the time of immigration. However, a different process is applied to those of Tibetan heritage, who must have a medical assessment within 30 days of arrival to rule out active TB, regardless of the results of their preimmigration assessment. This regulation was implemented due to data showing that Tibetan refugees had significantly increased rates of LTBI and active TB, even compared to other refugees. Between 1998 and 2001, 181 Tibetans arrived in Toronto, Canada, via the United States, of which 97 percent had a positive tuberculin skin test (TST) and 13 percent had active TB, and of those with active disease, 17 percent had multidrug resistant (MDR) TB strains [4]. A study comparing positive TST results between Tibetan and non-Tibetan refugees arriving in Minnesota between 1992 and 1995 found that 98 percent of Tibetan refugees tested positive [5]. This abundance of TB is further highlighted by 2012 data from the World Health Organization, which found the incidence of active TB in the Tibetan population within India to be approximately 645 per 100 000, compared to India's overall incidence of 176 per 100 000 [6].

Screening for LTBI is not a requirement for entry or resettlement; however there exists multiple primary care guidelines and published screening programs for targeted high-risk populations including refugees [1, 7, 8]. Canadian-based guidelines recommend screening all children and adult refugees under 50 years of age originating from countries with a high incidence of TB (>15 per 100 000) [1]. Foreign-born individuals who have other risk factors for reactivation TB (e.g., chronic kidney disease) are recommended to undergo screening for LTBI, regardless of age [1, 7]. The Center for Disease Control in the United States currently recommends that immigrants and refugees should be considered for LTBI screening using either TST or interferon gamma release assay (IGRA), although it also is not mandatory for resettlement [9].

China's occupation of Tibet has resulted in over 80 000 Tibetans being displaced. Many have settled in the impoverished Indian state of Arunachal Pradesh but Canada has recently released a national resettlement program that plans to offer visas to up to a thousand displaced Tibetans from this region before 2017. Hence, an estimated 400 Tibetan refugees arriving in Calgary, Alberta, will need to be assessed by Calgary TB Services. While earlier data suggests high rates of TB among Tibetan refugees, there is a paucity of data on current trends in TB and LTBI incidence among those arriving in Canada. In addition, there has been limited evaluation to demonstrate whether blanket screening all Tibetan refugees is preferable to targeted screening methods.

Our objectives were to determine the incidence of LTBI and active TB among newly arrived Tibetan refugees, as well as characterize the outcomes of current postlanding surveillance practices. We also determined whether there were any associations between preimmigration factors and TB infection.

## 2. Study Population and Methods

The study population included all Tibetan refugees referred by CIC for initial assessment at Calgary TB Services between 2014 and 2016. Currently, Calgary TB Services assess 100 percent of Tibetan refugees arriving in Calgary. All refugees were assessed by history, examination, and additional investigations including but not limited to chest radiograph, TST, IGRA (QuantiFERON®-TB Gold), and sputum acid-fast bacilli (AFB) smear and culture. As per current guidelines [1, 7], all refugees under 50 years of age were offered screening for LTBI using either TST or IGRA. Regardless of age, all refugees were assessed for active TB, in particular those with symptoms or abnormal chest radiograph. Further investigations for active TB were ordered as deemed appropriate by the clinician. Treatment with three months of rifampin and isoniazid was offered to patients with LTBI if there were no contraindications to treatment. Treatment acceptance was determined as verbal agreement between the physician and patient following discussion of treatment. Efforts to achieve treatment compliance were made by nurses and physicians through numerous means of communication (telephone, e-mail, and contacting the family physician). Treatment completion was defined as filling at least 75 percent of treatment doses. Patients were treated in accordance with current Canadian TB standards [7].

A retrospective chart review was performed to collect demographic factors including age, gender, date of arrival, medical comorbidities, and history of TB. Clinical outcomes including results of LTBI screening, sputum AFB smear and culture results, and treatment related information were also collected.

Statistical analysis was done using Stata/MP 14 software. Frequencies and percentages were calculated for categorical variables, and descriptive statistics, including means and standard deviation (SD), were obtained for continuous variables. Chi-square test or Fisher's exact test was used for categorical data analysis. We sought to determine if the following preimmigration factors were associated with LTBI or completion of treatment for LTBI: age below 30 years, gender, prior residence in a refugee camp, family history of TB, and smoking status. Associations were considered significant at *P* < 0.05.

Consent was waived given the retrospective nature of the study. Ethics approval was obtained from the Conjoint Health Research Ethics Board, University of Calgary.

## 3. Results

### 3.1. Demographics

A total of 180 Tibetan refugees were assessed by Calgary TB Services between 2014 and 2016. Baseline demographics are outlined in Table 1. All patients were privately sponsored refugees, meaning that they were sponsored for resettlement by Canadian citizens or private groups, arriving directly from the Arunachal Pradesh district in India. A history of previous pulmonary TB was found in 13 patients and all recalled receiving treatment, although the details were largely unknown. One patient had a history of nontuberculous mycobacterium (NTM) infection, another had TB lymphadenitis, and a third patient had TB uveitis in the past; all had treatment. None of the patients tested positive for HIV on preimmigration medical assessments.

### 3.2. Diagnosis of LTBI Testing

**Figure 1:**
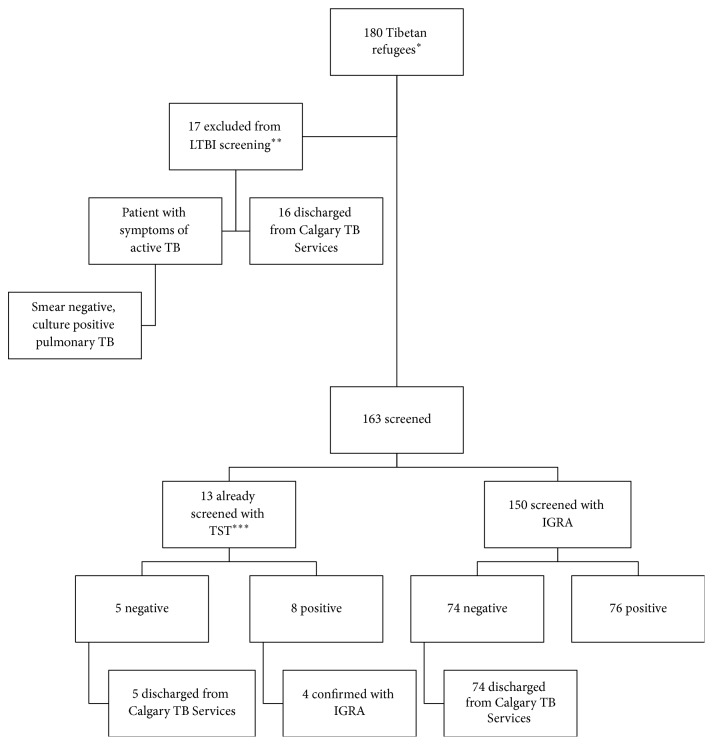
Screening for Tibetan refugees between 2014 and 2016. ^*∗*^100 percent of Tibetan refugees are assessed for active TB within 30 days of arrival in Canada at Calgary TB Services. ^*∗∗*^One patient was excluded for age over 50 years with no additional risk factors for reactivation TB, two patients had history of extrapulmonary TB, thirteen patients had history of treated pulmonary TB, and one patient had history of NTM infection treated with sufficient duration of rifampin to constitute treatment for LTBI. ^*∗∗∗*^Some patients had been assessed by Calgary Refugee Clinic or a primary care center where they had received LTBI screening with TST.

All patients under the age of 50 years without history of treatment for LTBI, active TB, NTM, or extrapulmonary TB were screened for LTBI with IGRA or TST (see Figure 1). Seventeen patients were excluded from LTBI testing, one patient with age over 50 years and no additional risk factors for reactivation TB, two patients with history of treated extrapulmonary TB, thirteen patients with history of treated pulmonary TB, and one patient with history of NTM infection treated with sufficient duration of rifampin to constitute treatment for LTBI. A total of 80 patients (49 percent) tested positive. Six patients were tested with both IGRA and TST, because the local refugee clinic had discovered a positive TST that was then confirmed by IGRA in our clinic setting. All cases of dual testing were concordant; four tested positive by TST only (patients declined confirmatory IGRA testing) and 70 tested positive by IGRA only. Age equal or greater than 30 years was associated with a diagnosis of LTBI (see Table 2).

### 3.3. Initiation and Completion of LTBI Treatment

**Figure 2:**
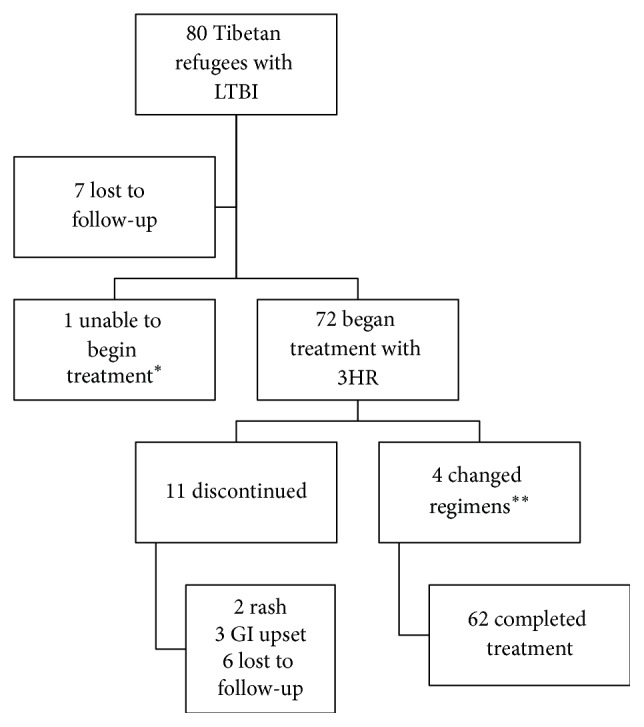
LTBI treatment between 2014 and 2016. 3HR = rifampin and isoniazid for three months. ^*∗*^Due to elevated liver enzymes. ^*∗∗*^Alternate regimens were either rifampin for four months or isoniazid for nine months.

Figure 2 characterizes treatment outcomes. Seventy-two of 79 treatment eligible patients accepted the offer of treatment, which was three months of daily isoniazid and rifampin. Eleven patients discontinued treatments after starting, all within the first month of treatment. Two patients discontinued treatment due to rash and another three patients due to abdominal pain. Six patients were lost to follow-up during treatment. Four other patients had to be changed to different regimens (rifampin for four months, or isoniazid for nine months) due to intolerance or liver enzyme elevation but completed treatment. In total, 76 percent of patients with LTBI completed treatment. Differences in age and gender were not associated with treatment completion.

### 3.4. Active TB

All 180 refugees were screened for evidence of active TB based on symptom history, chest radiograph, and, in some cases, sputum AFB smear and culture. One patient was found to have active pulmonary TB. He had a history of pulmonary TB fourteen years previously that was treated with isoniazid, rifampin, pyrazinamide, and ethambutol for one year. He had symptoms of cough at his initial appointment. Chest radiograph demonstrated fibronodular changes in his left upper lobe. Sputum was negative on AFB smear but culture grew* Mycobacterium tuberculosis*. Given his history of previous TB treatment and high rates of MDR TB previously identified in this population, he was empirically treated for multidrug resistant TB using isoniazid, rifampin, ethambutol, pyrazinamide, levofloxacin, and amikacin. Following the result of culture susceptibilities demonstrating pan-sensitive TB, this was later changed to isoniazid and rifampin for a total treatment duration of nine months.

## 4. Discussion

In this study, there was a single case of active TB found among a cohort of 180 Tibetan refugees resettling in Calgary, Canada. Of note, he had a history of TB and an abnormal chest radiograph before arrival; therefore his case of pan-sensitive TB would have been captured using the standard postlanding surveillance procedures applied to other refugee groups. If Tibetan refugees were screened similarly to refugees from other regions, then the total number of Tibetan refugees who would have undergone screening at Calgary TB Services would have been 60, including the active case. As it were, 180 Tibetan refugees underwent mandatory screening between 2014 and 2016.

LTBI was diagnosed in 49 percent of the population, substantially less than previously quoted rates of 97-98 percent [4, 5]. Of note, TST results in previous studies may have been falsely positive in the context of routine bacillus Calmette-Guérin (BCG) vaccination occurring within India. Improved living conditions as well as programs such as TB REACH [10] may be leading to lower rates of disease over the past decade. However, this is still higher than rates in other refugee populations and our data suggest that evaluation of Tibetan refugees is still a necessary component to enhanced TB case-finding strategies. A cross-sectional analysis of culture-confirmed TB cases reported to the US National TB Surveillance System used genotyping and epidemiological data to conclude that four out of five TB cases among foreign-born persons were attributable to reactivation TB [11]. Identification and treatment of LTBI should be prioritized in high-risk populations such as refugees originating from countries with high TB incidence.

To our knowledge, this is the first study to examine risk factors for LTBI among Tibetan refugees. We found that the diagnosis of LTBI was significantly associated with age greater than 30 years. A study of asylum seekers in Switzerland also found that the risk of LTBI increases with age [12]. An abnormal chest radiograph either before or after arrival almost met statistical significance for association with LTBI. Regardless, national guidelines currently recommend screening all refugees under 50 years of age from countries with high incidence of TB soon after arrival in Canada for LTBI and treating accordingly [1]. Currently, all refugees are assessed at a centralized refugee clinic in Calgary soon after arrival where LTBI screening is part of a routine assessment. Refugees who test positive for LTBI are then referred to Calgary TB Services for consideration of treatment. Therefore, the mandatory screening of all Tibetan refugees at Calgary TB Services is redundant and associated with increased cost and time. Tibetans are also the only refugee group subject to mandatory universal screening for TB after arrival, which is problematic for many reasons including additional clinic visits and stigma associated with TB.

Compliance rates for undergoing testing for LTBI were 100 percent, and this may be due to the utilization of IGRA for the majority of patients, which requires a single point of contact for completion. Various studies have shown that IGRA has at least equivalent sensitivity and greater specificity than TST, although Canadian-based guidelines recommend either TST or IGRA [7, 13]. In this cohort of patients, our practice is to use IGRA over TST in order to improve compliance via fewer visits and greater test simplicity [13]. Patients who were tested with TST in this cohort were either seen at the Calgary Refugee Clinic first or preferred a TST. Similar to the screening program in other cities, we offer confirmatory IGRA following positive TST [8]. Whether our local practice of using IGRA for LTBI screening is cost-effective remains to be determined. There were some difficulties in reaching patients following a positive result, mainly due to their relocation. Among those who returned to discuss treatment for LTBI, there was unanimous acceptance of treatment. However, several patients did not return for medication pickup. Further exploration of patient perceptions of possible inconveniences or risks of undergoing treatment may be helpful. A TB clinic in Seattle compared the use of cultural case management strategies to standard practice in three groups of refugees and found increased rates of acceptance and completion of LTBI treatment among all groups [14]. Their intervention involved bilingual staff members and trained community outreach workers that led to an increase in treatment completion rates among refugees from 37 to 82 percent (*P* < 0.001).

Our treatment completion rate was quite high at 76 percent. One factor may be the use of a three-month treatment regimen for the vast majority of patients, which is supported by previous reports showing higher rates of treatment completion with shorter treatment regimens [15, 16]. A trial demonstrated that patients randomized to three months of rifampin and isoniazid were 2.5 times more likely to complete treatment than those prescribed six months of isoniazid [17]. A meta-analysis comparing short course therapy with rifampin plus isoniazid to standard therapy with isoniazid found equivalence in terms of effectiveness and safety [18]. We observed a 12.5 percent incidence rate of adverse events requiring discontinuation of treatment among those treated with three months of isoniazid and rifampin, namely, rash, abdominal upset, and mildly elevated liver enzymes. There were no serious adverse events or deaths within the cohort. Age and gender did not impact rates of treatment completion in this study. Evidence for directly observed therapy (DOT) in LTBI treatment is inconsistent and not currently done at Calgary TB Services [19].

Also, all patients who defaulted on treatment did so within one month of starting treatment. This supports the notion that poor adherence during the first month of therapy is associated with a lower likelihood of treatment completion. A prospective trial by Menzies et al. suggested that targeted interventions be tailored for patients with suboptimal adherence during the first month [20]. Our experience is that refugees often relocate for potential employment, making it difficult to continue treatment without accurate contact information. There is evidence demonstrating that patients are frequently lost at each stage of screening, diagnosis, and treatment. A systematic review found that factors associated with fewer losses included having immunocompromised conditions as the indication for screening, being part of contact investigations and use of rifamycin-based regimens for treatment [21].

Our study has several limitations. Given the retrospective nature, details surrounding previous TB treatments and risk factors for infection could not be verified. Data on smoking, comorbidities, and other historical points were also self-reported, which may have affected accuracy. Also, certain comorbidities are likely to be undiagnosed (e.g., diabetes, renal disease) due to limited access to care in India. Also, study participants had often not yet been assessed by the refugee clinic before being seen at Calgary TB Services, due to the requirement to be assessed within 30 days of arrival. Finally, our sample size may not have been large enough to detect other associations with LTBI diagnosis or treatment completion.

## 5. Conclusion

In order to achieve the ambitious goal of ending the global TB epidemic, low-incidence countries must concentrate their efforts on more effective TB prevention. Our study brings into question the mandatory universal screening of incoming Tibetan refugees, as it results in a duplication of efforts with other primary care facilities providing routine health care for refugees with no evidence of enhanced case detection. Our data does not justify the current requirement by CIC for universal screening of Tibetan refugees arriving in Canada for active TB, compared to targeted postlanding surveillance for refugees from other regions.

## Figures and Tables

**Table 1:** Demographic information (*N* = 180).

Characteristic	*N* (%) or mean (SD)
Male gender	88 (49%)
Age at arrival, years	26.52 (11.09)
Confirmed refugee camp residence	115 (64%)
History of TB	13 (7.2%)
History of extrapulmonary TB	2 (1.1%)
History of NTM infection	1 (0.5%)
Family history of TB	5 (2.8%)
Smoker	18 (10%)
Previous smoker	8 (4.4%)

**Table 2:** Associations between demographic characteristics and diagnosis of LTBI.

	Number with LTBI	*P* value
Age less than 30 years	51/119	*P* = 0.009
Age 30 years or older	29/44	
Male	45/83	*P* = 0.181
Female	35/80	
Residence in camp	49/106	*P* = 0.320
No/unknown camp residence	31/57	
Smoker or prior smoker	16/25	*P* = 0.105
Never smoked	64/138	
Abnormal chest radiograph	39/68	*P* = 0.074
Normal chest radiograph	41/95	
Family history of TB	2/5	*P* = 0.517^*∗*^
No family history	78/158	

^*∗*^1-sided Fisher's exact test.
